# High prevalence of lack of knowledge of symptoms of acute myocardial infarction inPakistan and its contribution to delayed presentationto the hospital

**DOI:** 10.1186/1471-2458-7-284

**Published:** 2007-10-09

**Authors:** Muhammad S Khan, Fahim H Jafary, Azhar M Faruqui, Syed I Rasool, Juanita Hatcher, Nish Chaturvedi, Tazeen H Jafar

**Affiliations:** 1 Clinical Epidemiology Unit, Department of Community Health Sciences, Aga Khan University, Karachi, Pakistan; 2 Section of Cardiology, Department of Medicine, Aga Khan University, Karachi, Pakistan; 3 National Institute of Cardiovascular Diseases, Karachi, Pakistan; 4 National Heart and Lung Institute, Imperial College London, UK

## Abstract

**Background:**

We conducted an observational study to determine the delay in presentation to hospital, and its associates among patients experiencing first Acute Myocardial Infarction (AMI) in Karachi, Pakistan.

**Methods:**

A hospital based cross-sectional study was conducted at National Institute of Cardiovascular Disease (NICVD) in Karachi. A structured questionnaire was used to collect data. The primary outcome was delay in presentation, defined as a time interval of six or more hours from the onset of symptoms to presentation to hospital. Logistic regression analysis was performed to determine the factors associated with prehospital delay.

**Results:**

A total of 720 subjects were interviewed; 22% were females. The mean age (SD) of the subjects was 54 (± 12) years. The mean (SE) and median (IQR) time to presentation was 12.3 (1.7) hours and 3.04 (6.0) hours respectively. About 34% of the subjects presented late. Lack of knowledge of any of the symptoms of heart attack (odds ratio (95% CI)) (1.82 (1.10, 2.99)), and mild chest pain (10.05 (6.50, 15.54)) were independently associated with prehospital delay.

**Conclusion:**

Over one-third of patients with AMI in Pakistan present late to the hospital. Lack of knowledge of symptoms of heart attack, and low severity of chest pain were the main predictors of prehospital delay. Strategies to reduce delayed presentation in this population must focus on education about symptoms of heart attack.

## Background

Outcomes of patients presenting with acute myocardial infarction (AMI) are highly dependent on the prompt administration of reperfusion therapy, be it thrombolysis or primary percutaneous coronary intervention [1]. This dependency of outcomes on time is particularly applicable to thrombolytic therapy, which is, by far, the most common mode of reperfusion in acute myocardial infarction (AMI) owing to its widespread availability and ease of use. A large body of literature exists on the benefits of early administration of thrombolysis in AMI [2-4]. The mortality reduction is greatest when thrombolytic agents are administered within the first four hours after the onset of symptoms [5,6], particularly within the first 70 minutes, wherein the mortality reduction approaches 50% [7]. On the other hand, the benefits of thrombolytic therapy rapidly decline beyond six hours after symptom onset [7], and although some benefits may be observed as late as 12–24 hours after symptoms [8,9], the magnitude of the benefit is considerably smaller.

A critical prerequisite for early reperfusion, of course, is the patient presenting to the hospital promptly after the onset of symptoms, followed by administration of therapy in a timely manner. The time lapsed between the patient presenting to the emergency room and receiving the injection of thrombolytic therapy has been substantially reduced over the years to approximately 30 minutes in the United States [10]. A study conducted in Karachi, reported that the mean delay time foradministration of throbmolytics since arrival to ED was approximately 120 minutes [11]. However, in many countries prehospital delay on the part of the patient remains a substantial problem with almost half presenting more than 4 hours after symptom onset [11-13]. An analysis of temporal trends suggests that this prehospital delay has not changed substantially over time despite existing educational programs and the widespread availability of ambulance services for transportation to the hospital [12]. Factors associated with delayed presentation include increased age [12,14,15], female gender [12,14,15], ethnicity (blacks compared to whites) [12,14,15], medical insurance (private insurance compared to self pay) [16] and a prior history of angina, diabetes or myocardial infarction [15-17].

People of Indo-Asian origin have a high burden of coronary artery disease (CAD) and the latter is now the leading cause of death in the Indo-Pakistan subcontinent [18,19]. Although it is generally perceived that patients with AMI present relatively late in the Indo-Pakistan subcontinent, there is a paucity of data on patients in this part of the world. In one small study on 133 patients with AMI in an urban community hospital in India, Rajagopalan and colleagues observed that 36% of the patients delayed presentation to the hospital by more than 6 hours [20]. Unavailability of transportation, prior treatment by local practitioners, and lack of knowledge of symptoms contributed to this delay [13]. However, larger scale studies from Indo-Pakistan on health seeking behaviour of patients with AMI are lacking. The objectives of this study were to determine the extent of delay in seeking early hospital presentation after onset of AMI symptoms among patients suffering from their first AMI at a tertiary care hospital in Pakistan. We also sought to examine the factors associated with prehospital delay.

## Methods

A hospital based cross-sectional study was conducted in Karachi from July 2003 to February 2004 at the National Institute of Cardiovascular Disease (NICVD) Karachi, which although a tertiary care public hospital, is the initial point-of-care for a large majority of patients with heart disease from all socioeconomic strata in Karachi. The study population was defined as patients presenting with symptoms of AMI leading to the diagnosis of their first AMI and surviving the initial 24 hours. AMI was defined using the European Society of Cardiology and American College of Cardiology's criteria [21,22]. The presence of at least two of the following three factors was considered as diagnostic for AMI.

1. Typical chest pain lasting for at least 20 minutes,

2. Electrocardiogram showing ST elevation of at least 2 mm in two or more contiguous leads with subsequent evolution of the ECG, and

3. Diagnostic cardiac marker (doubling of creatine kinase with at least 10% MB fraction) or elevated or positive troponin I or T.

Ethical approval was given by the ethical review committee of Aga Khan University, and permission to conduct the study was given by the selected study hospital. The admission records of the Emergency Room (ER) of NICVD were examined daily. Every patient admitted to the hospital with a first AMI and surviving the initial 24 hours was screened for eligibility of the study. Eligible patients were approached by a trained research medical officer to seek informed consent. Patients who were conscious, and gave informed consent were invited to participate in the study. Patients with life threatening conditions, those who were intellectually impaired or developmentally delayed or were already admitted to the hospital at the time of onset of symptoms of AMI were excluded from our study.

### Primary Outcome

Delay was said to have occurred if the time interval from the onset of symptoms to presentation to the hospital was greater than or equal to six hours. We chose this time cut-off because evidence suggests that the benefits of thrombolytic therapy declines beyond 6 hours following presentation [7,23-25]. Our interest was to determine subjects who "miss" this therapeutic window and hence are delayed in presentation. Time variables were calculated as follows: the time of onset of symptom of AMI was determined as closely as possible by questioning the patient as well as family members living with the subject. The time of presentation to the hospital was documented in the notes upon first contact at the hospital. Time of presentation was derived by subtracting the former from the latter.

A structured questionnaire was used to collect data. Although not validated, different components of our questionnaire were derived from published studies [26-29]. Moreover, the content validity of questionnaire items was examined by clinical experts and peer review.

Subjects were asked about information on socio-demographicfactors: age, gender, ethnicity [30] marital status, level of formal education (defined as a person who had ever attended school), type of family system (nuclear family system was defined as a household consisting of two parents and their legal children; extended family system was defined as a household where multiple generations of family were living together), income and occupation.

For information on clinical characteristics, subjects were asked about history of hypertension, history of diabetes and general health behaviors i.e. tobacco use, physical activity and number of visits to any health care facility during past one year. Severity of pain was assessed using visual analog scale (VAS) [0 (no pain) to 10 (the worst pain)]. Patients with VAS pain scores of 0–4 were categorized as having mild pain, those with scores of 4–7 as moderate pain, and those with scores of 8 or more as having severe pain. Study subjects were asked about the symptoms of heart attack in an open ended fashion in order to permit unaided recall and four possible cardiac symptoms were identified – chest pain, palpitations, shortness of breath and "ghabrahat". The latter is a commonly described (yet poorly documented) symptom noted by patients presenting with acute coronary syndromes in Pakistan. "Ghabrahat" (fidgetiness) is a cluster of symptoms that includes a vague sense of anxiety, restlessness uneasiness and feeling of doom. Those who could identify at least one of these four symptoms of heart attack were arbitrarily defined as knowledgeable. Actual symptoms of AMI were asked after the initial interview. Subjects who sought medical care after six hours were questioned in open-ended fashion about possible reasons for the delay in seeking medical attention.

Frequency distributions, means (SD) and median (IQR) were calculated for all continuous variables and frequencies for categorical variables. Simple logistic regression analysis was run to investigate the relationship of each independent variable with delay. Variables with a p value of ≤ 0.25 in the univariate analysis (or of high biological importance, for example age) were entered into the multiple regression model. Multiple logistic regression analysis was applied to select the variables independently associated with delay in seeking early medical care among subjects with AMI. A p value of < 0.05 was considered statistically significant. Odds ratios and 95% confidence intervals were calculated to interpret our final model. The analysis was also repeated with using a cut-off of 2 hours to define delayed presentation. The purpose of this analysis was to see how sensitive our findings are to using a different cut off of our outcome variable.

## Results

**Figure 1:**
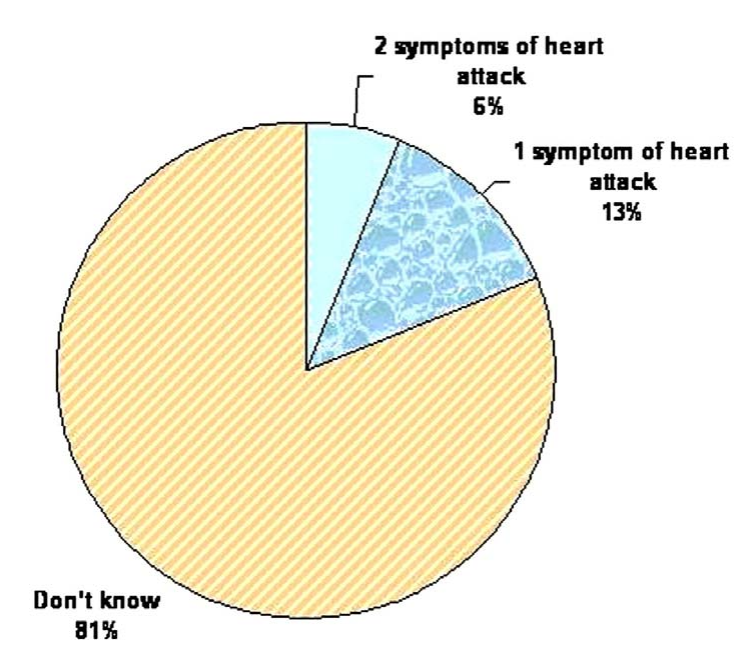
Distribution of knowledge of symptoms of heart attack among subjects with first AMI in Karachi, Pakistan.

A total of 720 patients with first AMI were stable enough to be interviewed, and were invited to enroll in the study. All 720 subjects consented. Seventy eight percent of the subjects were male and the mean age (SD) of the cohort was 54 (± 12) years. The median (IQR) times to presentation were 3.04 (6.0) hours. Almost all subjects reported chest pain (98%) and ghabrahat (fidgetiness) (94%) at the time of heart attack. Thirty-four percent of the study subjects sought medical care six hours or more after the onset of AMI time, that is, had a delayed presentation. Moreover, only 36% of study subjects sought medical care within two hours of onset of symptoms of AMI. Two-thirds (66%) of the study sample failed to recognize the symptoms of myocardial infarction (Table 1). Figure 1 shows that 81% of the study participants were not aware of any symptoms of heart attack, and only 6% could identify two or more symptoms of heart attack.

**Table 1:** Characteristics of the subjects with first acute myocardial infarction in a tertiary care hospital Karachi, Pakistan

Variable	Delayed*		Not delayed		P-value
		
	**n** **244**	**%** **33.9**	**n** **476**	**%** **66.1**	
Age					0.434
Younger ≤ 45 years	62	25.4	134	28.2	
Older > 45 years	182	74.6	342	71.8	
Sex					0.213
Male	197	80.7	365	76.7	
Female	47	19.3	111	23.3	
Marital status					0.715
Married	220	90.2	425	89.3	
Single	24	9.8	51	10.7	
Formal education					0.151
Yes	160	65.6	286	60.1	
No	84	34.4	190	39.9	
Ethnicity					0.987
Urdu	179	73.4	345	72.5	
Sindhi	20	8.2	41	8.6	
Punjabi	14	5.7	30	6.3	
Other§	31	12.7	60	12.6	
Type of family system					0.546
Nuclear	87	35.7	159	33.4	
Joint	157	64.3	317	66.6	
Ownership of television					0.109
Yes	136	56.0	294	62.2	
No	107	44.0	179	37.8	
Family history of heart disease					0.895
Yes	89	36.5	176	37.0	
No	155	63.5	300	63.0	
Distance from hospital (in KM)					0.195
< 10 Km	65	26.6	149	31.3	
≥ 10 Km	179	73.4	327	68.7	
Employment					0.286
Yes	121	49.6	256	53.8	
No	123	50.4	220	46.2	
Smoking status					0.345
Never smoked	116	47.5	244	51.3	
Past or current smoker	128	52.5	232	48.7	
Exercise€					0.559
Yes	9	3.7	22	4.6	
No	235	96.3	454	95.4	
Income (in PKRS)					0.885
> 1000	15	6.1	32	6.7	
5000–10000	96	39.3	195	41.0	
< 5000	120	49.2	220	46.2	
Didn't reveal	13	5.3	29	6.1	
Severity of pain					<0.001
Severe	64	26.2	353	74.2	
Moderate	85	34.8	68	14.3	
Mild	95	38.9	55	11.6	
Chest pain					0.650
Yes	237	97.1	465	97.7	
No	7	2.9	11	2.3	
Ghbrahat (Uneasiness)					0.339
Yes	232	95.1	444	93.3	
No	12	4.9	32	6.7	
Shortness of breath					0.406
Yes	215	88.1	429	90.1	
No	29	11.9	47	9.9	
Palpitation					0.005
Yes	192	78.7	413	86.8	
No	52	21.3	63	13.2	
Diaphoresis					0.040
Yes	224	91.8	439	92.2	
No	20	8.2	37	7.8	
Known Hypertension					0.840
Yes	107	43.9	205	43.1	
No	137	56.1	271	56.9	
Known Diabetes mellitus					0.893
Yes	66	27.0	131	27.5	
No	178	73.0	345	72.5	
Knowledge of modifiable risk factors					0.004
Yes	31	12.7	103	21.6	
No	213	87.3	373	78.4	
Knowledge of symptoms of heart attack					0.004
Yes	31	12.7	103	21.6	
No	213	87.3	373	78.4	

* Time interval from the onset of symptoms to presentation to the hospital was ≥ 6 hours

§ include Rajistani, Bangoli, Gujrati, Marhwi, Katchi, Sirayki or Hindi

€ Exercise (that lasted for at least 20 minutes with hard breathing and sweating for at least 3 days/week)

Table 2 shows the results of the multivariable analysis. Subjects with no knowledge of symptoms of heart attack were 1.82 times (95%CI; 1.10, 2.99) more likely to delay in seeking early medical care than those with more knowledge of symptoms of heart attack. Subjects with mild chest pain were much more likely to present late (OR 10.05 [95%CI; 6.50, 15.54]). These estimates were adjusted for age and income.

**Table 2:** Adjusted Odds Ratios and 95% CI of factors associated with delay in seeking early medical care among AMI^1 ^subjects in a tertiary care hospital Karachi, Pakistan

Variable	Delayed		Not delayed		Adjusted^2 ^OR	(95% CI^3^)	P-value
				
	**n**	**(%)**	**n**	**(%)**			
Knowledge of symptoms of heart attack							0.020
Yes^4^	31	(12.7)	103	(21.6)	1.00	-	
No	213	(87.3)	373	(78.4)	1.82	(1.10, 2.99)	
Severity of Pain (using Visual Analog Scale (0–10))							<0.001
Severe	64	(15)	353	(85)	1.00	-	
Moderate	85	(56)	68	(44)	6.86	(4.50, 10.47)	
Mild	95	(63)	55	(37)	10.05	(6.50, 15.54)	

^1^AMI = Acute Myocardial Infarction;

^2^adjusted for age, and income

^3^CI = confidence interval

^4 ^A person who knows at least one symptom of heart attack

The same factors were identified in a sensitivity analysis using a cut off of 2 hours for defining delay as in the main analysis. However, we also found that those who did not have access to television (40%) had significantly greater odds (95% CI) 2.05 (1.06 to 3.97) for delayed presentation.

## Discussion

The primary aim of the study was to determine the extent of delay in presentation to the hospital among patients suffering from AMI in Pakistan. We also sought to examine the independent correlates of this delay. The results of this study demonstrate that over one third of the study population sought medical care six or more hours after the onset of symptoms of AMI. Lack of knowledge of symptoms of heart attack and less severe chest pain were significantly associated with delayed presentation.

Cardiovascular diseases have emerged as a major health burden in developing countries [31]. In the year 2003, 16.7 million people died from CVD, accounting for 30.3% of all deaths worldwide [32]. More than half of CVD deaths were in developing countries. The population of South Asia (Pakistan, India, Bangladesh, Nepal and Sri Lanka) represents more than a quarter of the developing world, and is likely to be strongly affected by the increase in CVD [33]. While programs for primary prevention of CVD in these countries are vital, measures to ensure delivery of equitable care for those who have these diseases are equally important. At least a third of the patients with AMI presented to the hospital after six hours of delay. These patients were at increased risk from adverse outcomes. We also found that lack of knowledge of symptoms of heart attack was significantly associated with delay. Moreover, it was rather disappointing to note that 81% of the study participants were not aware of any symptoms of heart attack, and only 6% could identify two or more symptoms of heart attack. Consistent with this, two-thirds of the study cohort failed to recognize that they were suffering from a myocardial infarction. Clearly lack of knowledge of symptoms appears to translate to behavioral deficiencies culminating in failing to seek medical attention early for AMI. This is consistent with other studies [13,34,35] and serious effort needs to be put into educating the public on the symptoms of AMI. These findings indicate a need for inclusion of public awareness of signs and symptoms of heart diseases in national level programs for prevention of CVD.

In the current study, the median time (IQR) to presentation was 3.0 (6.0) hours. This finding is consistent with other studies [13,20,36] although the range of presentation times reported in the literature is fairly wide [36-38] probably reflecting the differences in study populations and patient characteristics. That said, our data somewhat refute the perception that most Indo-Asian patients, for a variety of reasons, seek medical assistance later than those in the West. Moreover, we found the median distance between subject's homes to cardiac hospital was 16 kilometers, and distance was not associated with delay in the final model. Nevertheless, due to the resource scarce environment, poor public health care infrastructure, and affordability issues surrounding cost of medications, the outcomes of patients with AMI who present late are probably worse in developing countries. Therefore, greater efforts are needed to prevent such delays in these countries.

We found a dose response relationship of severity of pain and time that lapsed in early seeking of medical care. This finding is in line with other studies [20,39-41]. A shorter delay in patients with severe pain could be due to the symptoms that are themselves severe enough that stimulate the patient to seek help.

Older age [16,20,34,36,42-46], female gender [34,36,43,47] and low socioeconomic status [43] have been identified as predictors of delay in patients with myocardial infarction in earlier studies but these factors were not found to be significant in our study. Possible reasons for this include the relatively small number of females (158), and the uniformity in socioeconomic status with the vast majority (>90%) belonging to the lower middle class with monthly income of less than US $150.

Our study has some limitations. Our study does not represent the patients with AMI who die at home or en-route to the hospital. However, in a substantial number of these patients, the first symptom of their myocardial infarction is sudden death and the question of time to presentation is irrelevant. In a smaller subset of patients who do develop chest pain prior to out-of-hospital-death it is conceivable that the latter may have been circumvented had the patient presented earlier to the hospital. This relationship (if any) could not be captured by our study. Only patients who survived the first 24-hours of the index event and were clinically stable to give an interview were enrolled in the study. This introduces the possibility of a selection bias as those excluded due to mortality in the initial 24-hours or critical illness or subjects with no chest pain were the ones most likely to have more significant delay than the remaining cohort. Thus our evaluation of the correlates of delayed presentation may be somewhat biased. However, it was necessary to exclude these sick patients as they would have been unable to give a complete interview. Therefore, it is possible that the true proportion of patients who delay seeking appropriate care is somewhat higher than reported in our study. However, it is likely that the correlates we identified would have been shared by the subgroup we were unable to capture.

As the study cohort comprises of patients experiencing their first infarction, these results may not be extrapolated to those with more established heart disease. Although total delay in presentation was studied, we did not dissect out the intricacies of this delay including decision time and potential time lost by initial consultation with the patient's general practitioner, the latter being fairly common in this country. Thus we are unable to provide more insight into the break-up of individual patients' delay in seeking care. The number of potential symptoms of heart attack identified by patients was limited. This was due to the open ended mode of questioning them, which we undertook to assess unaided recall. Many symptoms, including radiation to the arms, neck, jaw etc. were not mentioned by patients as possible symptoms of heart attack, emphasizing the lack of knowledge. It is, of course, possible that providing cues to aide recall may have led to a greater estimate of knowledge. Furthermore, the use of "ghabrahat" (fidgetiness) as a symptom of myocardial infarction is based on local experience but is poorly documented in the literature. The use of one out of four symptoms as a cut-off for knowledgeable is arbitrary; that said, under 20% were able to describe one or more symptom. The use of an unvalidated questionnaire carries its own set of limitations; however, the questions were derived from the published literature and were ratified by local experts and peers. Finally, the study was performed in an urban city and findings may not be generalizable to rural Pakistan. However, one can surmise that patients in rural areas probably present even later when they experience an AMI.

Strengths of our study include the site of study which was based at the largest public hospital for heart disease serving the population of urban metropolitan city of Karachi, the prospective study design with inclusion of all eligible consecutive cases, and the 100% response rate.

## Conclusion

In conclusion, over one-third of patients suffering from a first AMI in urban Pakistan present beyond 6 hours following the onset of symptoms. The vast majority of subjects failed to recognize symptoms of AMI. Factors associated with delayed presentation include lack of knowledge of symptoms of AMI and less severe chest pain. Indo-Asian countries are facing an epidemic of cardiovascular disease, yet the level of recognition of symptoms is unacceptably low, and consequently leads to significant delay in seeking treatment. Strategies to reduce this delay in this population must focus on education on the diversity of coronary artery disease symptoms and benefits of presenting promptly to the hospital. Studies have also suggested that knowledge and mass public education campaigns alone may not be sufficient to change behaviour in seeking treatment for ACS symptoms [48-50]. The impact of other strategies including targeted education of high risk groups and their family members, content of educational message in particular emphasizing the importance of benefit of administration of early administration of thromolytics, and calling an ambulance for a suspected AMI need further study.

## Competing interests

The author(s) declare that they have no competing interests.

## Authors' contributions

MS did the study under the supervision of TJ and performed the statistical analysis, and drafted the manuscript. JH supervised the statistical analysis. FH, IR& AF provided the clinical knowledge of AMI. TJ, JH and NC contributed to review, and to the revision of the report. All authors read and approved the final manuscript

## Pre-publication history

The pre-publication history for this paper can be accessed here:


